# Exploring the Link between Anticoagulation, Cognitive Impairment and Dementia in Atrial Fibrillation: A Systematic Review

**DOI:** 10.3390/jcm13082418

**Published:** 2024-04-21

**Authors:** Abhimanyu Agarwal, Mohamed A. Mostafa, Muhammad Imtiaz Ahmad, Elsayed Z. Soliman

**Affiliations:** 1 Epidemiological Cardiology Research Center, Section on Cardiovascular Medicine, Department of Medicine, Wake Forest University School of Medicine, Winston Salem, NC 27157, USA; abhsworld@gmail.com (A.A.); mmostafa@wakehealth.edu (M.A.M.); 2 Department of Medicine, Section on Hospital Medicine, Medical College of Wisconsin, Wauwatosa, WI 53226, USA; imtiaz186@gmail.com

**Keywords:** atrial fibrillation, cognitive impairment, dementia, oral anticoagulants, antiplatelets

## Abstract

**Background:** The impact of oral anticoagulants (OACs) on cognitive impairment and dementia in patients with atrial fibrillation (AF) is not well characterized. This systematic review aims to address this knowledge gap. **Methods:** SCOPUS and PubMed searches were conducted to identify articles in the English language investigating the association between the use of OACs and cognitive impairment and dementia. We excluded non-original research studies and studies that did not report data on cognitive impairment or included patients who underwent open heart surgery or had psychiatric illnesses or cancer. **Results**: Out of 22 studies (*n* = 606,404 patients), 13 studies (*n* = 597,744 patients) reported a reduction in cognitive impairment/dementia in those undergoing thromboprophylaxis. Using direct oral anticoagulants (DOACs) was associated with a lower incidence of cognitive impairment in 10 studies (*n* = 284,636 patients). One study found that patients undergoing dual therapy (*n* = 6794 patients) had a greater incidence of cognitive impairment compared to those undergoing monotherapy (*n* = 9994 patients). Three studies (*n* = 61,991 patients) showed that AF patients on DOACs had a lower likelihood of dementia diagnosis than those on vitamin K antagonists (VKAs). Dementia incidence was lower when VKAs were under good control. **Conclusions**: The use of oral anticoagulants has the potential to prevent cognitive impairment and dementia in patients with AF. Since most of the published research on this subject is observational in nature, more randomized controlled trials are needed to fully understand the effect of anticoagulants on cognitive function.

## 1. Introduction

The prevalence of atrial fibrillation (AF) has been increasing worldwide, especially in the older population. It has been suggested as a risk factor for developing dementia and other cognitive problems even without a prior history of stroke [1,2]. The high prevalence and possible cognitive complications of AF pose a significant health and economic burden on the general population. Multiple mechanisms have been proposed as an explanation for the association between AF and cognitive impairment. This includes reduced blood flow, inflammation, and microemboli [1,3,4,5,6,7]. Shared risk factors such as old age, arterial hypertension, diabetes, hyperlipidemia, sleep apnea, coronary artery disease, heart failure, chronic kidney disease, obesity, inactivity, and excessive alcohol use [8,9,10,11] could also explain the link between cognitive impairment and AF. Therefore, it has been speculated that preventive strategies for AF complications, namely the use of anticoagulants, could also target cognitive impairment by lowering the risk of microemboli and stroke. However, excessive or prolonged anticoagulant usage may also raise the risk of brain microbleeds [12], which could negatively influence cognitive function. Therefore, further research is needed to understand the impact of medications used to prevent AF complications on cognitive impairment and dementia. In this systematic review, we investigated whether different oral anticoagulants, adherence to treatment and time in therapeutic range (TTR) impact cognitive impairment or dementia among patients with AF.

## 2. Methods 

In conducting this systematic review, we followed the methods outlined in the Preferred Reporting Items for Systematic Reviews and Meta-Analyses (PRISMA) [13].

### 2.1. Data Sources 

Systematic searches were conducted on the PubMed and SCOPUS databases from their inception until 20 June 2023, using specific search terms ‘atrial fibrillation’, ‘cognitive decline’, ‘cognitive impairment’, ‘cognitive function’, ‘dementia’, ‘atrial fibrillation treatment’, ‘anticoagulants’, ‘antiplatelet drugs’ and ‘thromboprophylactic drugs’.

Eligibility Criteria:

Only studies published in the English language were deemed eligible if they met the following criteria:

▪ Observational studies or randomized controlled trials.
▪ Studies enrolling patients with AF, either permanent, persistent or paroxysmal, and who are >18 years old.
▪ Studies enrolling patients with AF who are receiving anticoagulant therapy.
▪ Studies whose outcomes of interest are clearly reported and assessed via recognized scoring systems, e.g., MMSE (Mini Mental State Exam), MoCA (Montreal Cognitive Assessment), IQCODE (Informant Questionnaire on Cognitive Decline in the Elderly), ICD codes, etc.

Exclusion criteria:

▪ Studies enrolling patients who underwent open heart surgery or currently have underlying psychological disorders or cancer.
▪ Studies focusing on the pathophysiology of AF or that do not report data on cognitive impairment or only provide results from brain imaging, biomarkers, or genetic markers.
▪ Studies that do not include original data, or are editorials, case reports, case series, systematic reviews, or meta-analyses.

### 2.2. Study Identification

We reviewed articles based on their titles and abstracts, applying predetermined inclusion and exclusion criteria along with a standardized data collection form. Articles that did not meet the inclusion criteria based on the abstract were not considered for full-text review. The decision to include full texts was made through consensus. All findings were transferred to Zotero, an open source research tool used for organizing and analyzing data, and any duplicate entries were removed.

### 2.3. Data Extraction and Outcomes 

A structured form for data collection was used to obtain details from every study, such as the study’s design, patient demographics, initial variables, method of cognitive assessment, length of follow-up in years, AF detection methods, maximum adjusted variables, hazard risks (HR)/odds ratios (OR) with a 95% CI, and the treatment administered.

Our primary objective was to assess the effects of OACs on cognitive impairment in AF patients. Studies reporting the use of specific OACs, such as VKAs (warfarin), as well as more recent non-vitamin K-dependent novel oral anticoagulants (NOACs), were also included. In cases where the article did not identify them precisely, oral anticoagulants were collectively referred to as OACs.

### 2.4. Data Synthesis and Analysis

We refrained from conducting a meta-analysis due to notable variations in methodologies and heterogeneity among the studies we reviewed. These differences include the approaches used in conducting the studies, the characteristics of patients involved, the tools utilized for cognitive function assessment, the outcomes observed, and the statistical techniques employed. This rendered the pooling of data unsuitable and potentially prone to generating misleading conclusions.

### 2.5. Risk of Bias Assessment

The quality of each article was assessed using the Newcastle-Ottawa Scale (NOS) items and The Cochrane Collaboration’s tool for evaluating the risk of bias in randomized trials. Articles with an NOS score of ≥6 stars were classified as being of high quality, while those with an NOS score of <6 stars were classified as being of low quality.

## 3. Results 

**Figure 1:**
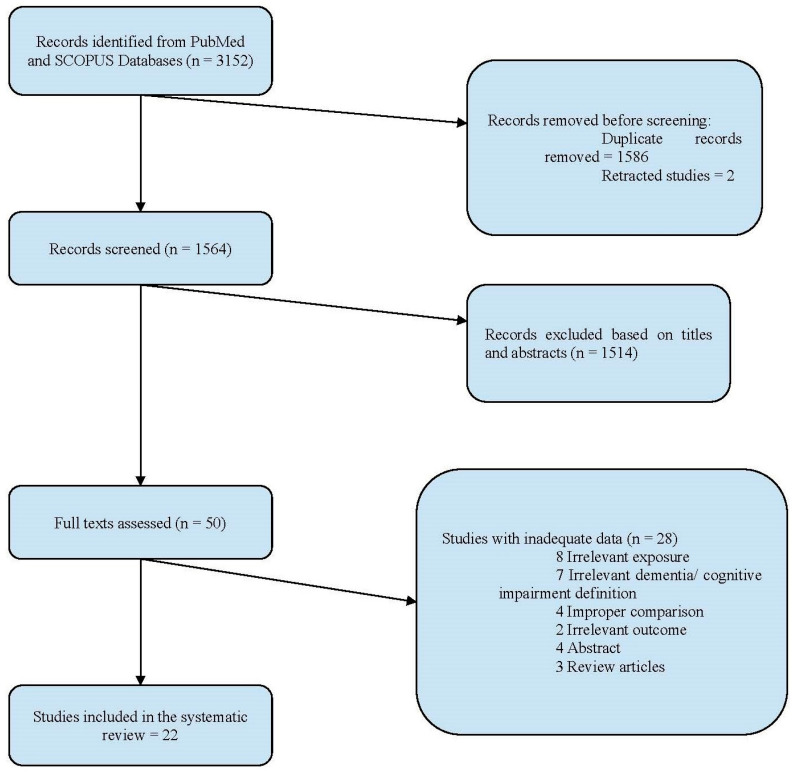
Study selection inclusion/exclusion criteria.

In a total of 22 studies (8 prospective cohort, 8 retrospective cohort, 3 RCT, and 3 cross-sectional) involving 606,404 patients, 39,432 patients were taking DOACs, 30,866 patients were on antiplatelet medication, and 262,940 patients were on VKA. DOAC usage was examined in 10 studies [14,15,16,17,18,19,20,21,22,23], VKA usage was examined in 21 studies [14,15,16,17,18,19,20,21,22,23,24,25,26,27,28,29,30,31,32,33,34], while antiplatelet use was assessed in 6 studies [14,15,24,25,26,35]. The time in therapeutic range (TTR) was assessed in 10 studies [18,22,24,26,28,29,30,32,33,34]. Additionally, ten studies primarily focused on the MMSE [14,16,18,19,25,27,29,30,34,35]. Figure 1 illustrates the criteria used to include or exclude studies in this review. Table 1 provides a summary of the characteristics of the included studies. A summary of the findings from the studies that examined the associations between oral anticoagulants and cognitive impairment and dementia in patients with AF is also outlined in Table 1. As shown in the table, the key findings are as follows:

### 3.1. Associations with Oral Anticoagulants

Three cohort studies [15,20,23] indicated that the use of OACs vs. no OACs was associated with a lower incidence of dementia/cognitive impairment. One report revealed a higher risk of cognitive impairment with dual therapy [15]. Additionally, one report showed that dual therapy was associated with a higher risk of dementia/cognitive impairment compared to no treatment (Table 1)**.**

### 3.2. Association with Vitamin K Antagonists

Three cohort studies [15,26,31] and one cross-sectional study [27] addressed this question. Overall, while some studies suggest a potential benefit of warfarin over non-OAC treatment and a reduction in dementia risk with DOACs compared to VKAs, other studies showed mixed or non-significant differences. Therefore, the takeaway is that the association between anticoagulant types and dementia/cognitive impairment risk remains complex, and further research is warranted (Table 1).

### 3.3. Direct Oral Anticoagulant vs. Vitamin K Antagonists

Four cohort studies [15,20,21,22] and two RCTs [18,19] provided evidence suggesting that DOACs may be associated with a reduced risk of dementia or cognitive impairment compared to VKAs in patients with AF. However, some studies did not find significant differences in dementia risk between DOACs and VKAs. Additionally, RCTs showed comparable incidence rates of dementia and stroke between DOACs and VKAs, with no significant differences in cognitive function scores between the two groups (Table 1).

### 3.4. Direct Oral Anticoagulant vs. Antiplatelet 

Two cohort studies [15,35] compared the use of DOACs with antiplatelet medications in relation to cognitive impairment in atrial fibrillation patients. Their findings suggest that DOACs may have a protective effect against cognitive impairment compared to antiplatelet medications in AF patients (Table 1).

### 3.5. Vitamin K Antagonist vs. Antiplatelet 

One cross-sectional study [24] and one RCT [25] found that warfarin, particularly when TTR is controlled well, may be more beneficial in terms of cognitive outcomes compared to aspirin in AF patients (Table 1).

### 3.6. Warfarin/VKAs and Time in Therapeutic Range (TTR): Evaluating the Relationship 

Four cohort studies [22,26,28,32], one cross-sectional study [24] and two RCTs [18,33] explored the relationship between warfarin/VKA usage and TTR in relation to cognitive impairment and dementia risk with varying statistical significance observed. Overall, the findings suggest that maintaining good control of INR levels, as reflected by a higher TTR, is associated with a decreased risk of cognitive impairment and dementia in AF patients receiving warfarin or VKA therapy. However, studies showed varying statistical significance regarding this relationship, with some reporting a significantly decreased incidence of dementia with good INR control (TTR > 70%) compared to poor INR control [22]. Additionally, AF patients with a TTR of 65% or above had a non-significantly decreased probability of developing new-onset dementia compared to those with a TTR below 65% [26]. Lower mean TTR percentages were associated with a higher risk of dementia, with decreasing TTR categories showing increased dementia risk [28,32]. Furthermore, using warfarin with a TTR below the median value was linked to a higher risk of cognitive impairment, and incrementally higher risks for dementia were observed as TTR worsened [24,33]. Overall, these findings emphasize the importance of maintaining good control of INR levels within the therapeutic range to mitigate the risk of cognitive impairment and dementia in AF patients receiving warfarin or VKA therapy (Table 1).

### 3.7. Association between Cognitive Impairment, Time in Therapeutic Range (TTR) and Anticoagulation Control 

Three cohort studies [29,30,34] suggested that cognitive impairment may impact anticoagulation control, potentially affecting the management of AF patients receiving VKAs. The findings indicate that lower MMSE scores were associated with lower TTR values, and patients with cognitive impairment tended to use more VKA-interacting drugs (Table 1).

### 3.8. Association of Non-Adherence to Antithrombotic Therapy and Medication Type with Cognitive Impairment 

One cross-sectional [14] and two cohort [16,17] studies showed that non-adherence to antithrombotic therapy was associated with cognitive impairment, while higher medication adherence, particularly with DOACs, may contribute to improved cognitive function in atrial fibrillation patients. Additionally, cognitive function itself can influence medication adherence, with better cognitive function associated with better adherence (Table 1).

## 4. Discussion

Based on this systematic review, we found that the use of OACs in patients with AF is generally associated with a lower risk of cognitive impairment. Only 1 out of the 22 studies included in our review demonstrated a detrimental association between thromboprophylaxis and cognitive impairment. No significant differences were seen between DOACs and VKAs, and both were more effective than antiplatelet medications. A lower risk of cognitive impairment was also linked to maintaining good control of TTR/INR levels. Notably, studies have shown that cognitive function may affect adherence to oral anticoagulants, which may increase morbidity in AF patients.

### 4.1. Mechanism of Cognitive Impairment and Dementia in AF 

The relationship between AF and dementia may be explained by shared vascular risk factors [36,37] and AF-induced silent brain infarcts and thromboembolic strokes [38,39,40,41,42,43,44]. Stroke, the most severe consequence of AF, is due to blood stasis in the left atrial appendage and subsequent clot formation, which ultimately propagates to intracranial vasculature, leading to cardioembolic stroke [45,46,47,48]. It is suspected that the association between micro-embolism and dementia is related to symptomatic strokes. However, most micro cerebral infarcts discovered using brain MRI do not manifest as apparent strokes [49]. This may explain why patients with dementia sometimes have no signs of a stroke. A previous report has shown that nearly one-third of patients with AF exhibit evidence of silent brain infarcts on MRI [50]. Anticoagulation can reduce the risk of subclinical strokes, hence potentially reducing the likelihood of cognitive impairment, as suggested by the results of our systematic review. 

Cerebral microhemorrhage appears to also be common among AF patients, regardless of whether they are administered OACs or not [51]. A meta-analysis involving approximately 7000 patients with AF revealed a prevalence of microbleeds reaching 28% [52]. These results are consistent with the proposed mechanism that subclinical cerebral ischemia lesions may increase the risk of dementia in patients with AF [36,49,53,54].

### 4.2. Impact of Oral Anticoagulants on Cognition and Dementia

Our findings of a favorable impact of anticoagulants on cognitive impairment and dementia agree with prior reports [36,44]. Similarly, lower TTR has been previously shown to increase the risk of dementia [55,56], as we reported. There is evidence that it is feasible to maintain cognitive function and lower the risk of dementia by properly regulating INRs within the therapeutic range [36]. Nevertheless, another report could not provide evidence of cognitive benefit or harm from anticoagulation [57].

It is worth noting that various factors could potentially influence the decision-making process regarding the use of oral anticoagulation, including frailty, increased fall risk, and bleeding diathesis. Additionally, some of these patients may have preexisting conditions that could independently elevate the risk of cognitive impairment, regardless of oral anticoagulation use [58].

### 4.3. Comparing Different Medications 

In our review, we found no significant differences between DOACs and VKAs, and both were more effective than antiplatelets. However, in a previous report [44], the antiplatelet treatment showed equivalent efficiency to anticoagulant therapy in preventing dementia among patients with AF. Differences in the characteristics of the patients enrolled in the studies may explain differences in the results. Since the use of anticoagulants in patients with AF is dictated by the guidelines to prevent stroke, it is a challenge to compare the impact of different anticoagulants and antiplatelets in isolation from the comorbidities of the patients.

As ablation has become increasingly popular as a mainstream treatment for patients with longstanding AF in recent years, it is vital to acknowledge its association with a risk of silent ischemic strokes and cerebral microbleeds, which could potentially lead to declining cognitive function in this population [59,60]. In our review, we focused on medications only. Future research is needed to investigate the impact of ablation on cognitive function in AF patients.

## 5. Conclusions

Our review highlights the potential benefit of oral anticoagulants in reducing the risk of cognitive impairment and dementia in patients with AF. Further research is needed to understand the complex relationship between AF with cognitive impairment and dementia, as well as the potential impact of anticoagulants on prevention.

## Figures and Tables

**Table 1:** Characteristics and outcomes of studies evaluating the association between anticoagulation and cognitive impairment among AF patients.

Article	Type of Study	Cognitive Evaluation Method	Age (Year)	Sample Size (n)	Male (%)	Treatment	Main Findings	Magnitude of Association
Bunch et al. [19], 2022	RCT	MMSE, ADAS, DAD	73.7	63	57.1	DOACs, VKAs	Lower incidences of dementia/cognitive decline with both DOACs and VKAs. No significant difference between the two.	MMSE after 24 months: Dabigatran: 28.8 ± 1.8 Warfarin: 28.4 ± 1.8
Caramelli et al. [18], 2022	RCT	MMSE, MoCA	75	149	60.4	DOACs, VKAs	No difference observed between DOACs and VKAs regarding the main outcome.	Difference D-W (95% CI): −0.12 (−0.88 to 0.63)
							No difference in change in cognitive scores from baseline according to TTR subgroups (<70% and ≥70%).	MMSE: Contrast (95% CI): −0.19 (−1.20 to 0.83) MoCA: Contrast (95% CI): 0.14 (−1.13 to 1.41)
Wong et al. [26], 2022	Retrospective Cohort	Not Mentioned	76.4	3284	51.6	VKAs, Antiplatelets	Lower incidence with VKAs vs. no VKAs.	0.14%/year vs. 1.04%/year
							No significant difference in dementia incidence between >65% TTR vs. <65%TTR.	0.16% per year vs. 0% per year
Cadogan et al. [22], 2021	Retrospective Cohort	Read Codes GP-recorded	76	39,200	55.4	DOACs, VKAs	Negative association with DOACs vs. VKAs.	HR (95% CI): 0.84 (0.73, 0.98)
							Negative association with >65% TTR.	HR (95% CI): 0.73 (0.57, 0.92)
Malavasi et al. [35], 2021	Prospective Cohort	MMSE	74	437	61.3	Antiplatelets	Positive association with cognitive impairment.	OR (95% CI): 4.352 (1.583, 11.963)
Mongkhon et al. [15], 2020	Prospective Cohort	Read Codes or Antidementia drugs	NA	84,521	NA	DOACs, VKAs, Antiplatelets	Negative association with warfarin vs. no OACs.	HR (95% CI): 0.90 (0.85, 0.95)
							Positive association with dual therapy vs. no treatment.	HR (95% CI): 1.17, (1.05, 1.31)
							Negative association with OACs vs. antiplatelet.	HR (95% CI): 0.84 (0.79, 0.90)
							No significant difference between DOACs and warfarin.	HR (95% CI): 0.89 (0.70, 1.14)
Petroni et al. [24], 2020	Cross Sectional	SPMSQ (≥5 errors)	80	212	45.0	VKAs, Antiplatelets	Negative association with antiplatelets vs. VKAs	OR (95% CI): 24.74 (1.27, 482.12)
							Lower incidence of cognitive improvement with VKAs compared to aspirin.	Incidence at 33 months: Warfarin: 13% vs. Aspirin 64%
							Negative association with below-median TTR vs. VKAs.	OR (95% CI): 21.71 (4.35, 108)
Field et al. [23], 2019	Retrospective Cohort	ICD-10 ^§^	70.1	15,276	61.2	DOACs, VKAs	Negative association with DOACs vs. no DOACs.	HR (95% CI): 0.87 (0.70, 1.08)
Seong et al. [14], 2019	Cross Sectional	MMSE	74.2	277	59.2	DOACs, VKAs, Antiplatelets	Negative association with OACs vs. no OACs.	HR (95% CI): 0.90 (0.85, 0.95)
Friberg et al. [20], 2018	Retrospective Cohort	ICD-10	74.8	444,106	55.3	DOACs, VKAs	Negative association with DOACs vs. no DOACs.	HR (95% CI): 0.52 (0.50, 0.55)
							Negative association with VKAs vs. no VKAs.	HR (95% CI): 0.62 (0.60, 0.64)
							No differences between DOACs and VKAs.	HR (95% CI): 0.97 (0.67–1.40).
Madhavan et al. [28], 2018	Prospective Cohort	ICD-9	71.2	2800	53.4	VKAs	Mean TTR percentage was lower in patients who developed dementia compared to over a year prior and those without dementia.	TTR (dementia): 51.6% ± 26.5% TTR (baseline): 52.2% ± 24.8% TTR (no dementia): 52.5% ± 26.7%
							Risk reduction was consistent, regardless of whether the increase in TTR percentage coincided with a decrease in time spent in the supratherapeutic range or the subtherapeutic range	Supra- vs. sub-therapeutic range: HR (95% CI): 0.67 (0.57, 0.79) vs. (HR (95% CI): 0.71, (0.64, 0.79), respectively
Bunch et al. [33], 2016	Retrospective Cohort	ICD-9, ICD-10	72.5	4460	53.5	VKAs	Multivariate adjusted risk for dementia was incrementally higher as TTR worsened.	TTR 51–75% vs. >75%: HR = 1.30, *p* = 0.10. TTR: 26–50% vs. >75%: HR = 1.57, *p* = 0.02. TTR: ≤25% vs. >75%: HR = 1.92, *p* = 0.005.
Gorzelak-Pabiś et al. [30], 2016	Prospective Cohort	MMSE	76	104	44.2	VKAs	Patients with lower MMSE scores had lower TTR values	MMSE ≥ 27: TTR = 61% ± 27% MMSE < 24: TTR = 28% ± 26% MMSE 24–26: TTR = 43% ± 23%
							Patients with cognitive decline used more VKA-interacting drugs.	52% vs. 39%
							TTR value and number of interacting drugs did not significantly correlate with MMSE score groups.	Normal MMSE: r = 0.01; *p* > 0.05 MMSE < 27: r = 0.15; *p* > 0.05
Jacobs et al. [21], 2016	Retrospective Cohort	ICD-9 ^§^	72.4	5254	59.0	DOACs, VKAs	Negative association with DOACs vs. VKAs	HR(95% CI): 0.49 (0.35, 0.69).
Jankowska-Polańska et al. [16], 2016	Prospective Cohort	MMSE	73.5	111	50.5	DOACs, VKAs	Higher cognitive function was independently linked to improved medication adherence in AF patients	β = 1.139, SE_β_: 0.093
Annweiler et al. [27], 2015	Cross Sectional	MMSE	83.4	267	43.1	VKAs	Negative association with VKAs by the backward stepwise model	OR (95% CI): 14.38 (1.57, 131.76)
							Crude risk difference of cognitive decline across types of VKAs exists based on use vs. non-use of:	
							Acenocoumarol	OR (95% CI): 0.26 (0.16, 0.69)
							Warfarin	OR (95% CI): 0.08 (0.15, 0.32)
							Fluindione	OR (95% CI): 0.15 (0.02, 0.28)
Horstmann et al. [17], 2015	Prospective Cohort	MoCA	72.9	160	62.3	DOACs, VKAs	Better adherence with DOACs vs. VKAs.	94.6% vs. 82.6%
							No difference in cognitive function between DOACs and VKAs at 12 months	Median MoCA for VKAs vs. DOACs: 24 vs. 25
Jacobs et al. [32], 2014	Retrospective Cohort	ICD-9	73.7	2605	54	VKAs	Higher dementia risk with worsening TTR range.	TTR ≤ 25%: HR 5.34, TTR 26%–50%: HR 4.10, TTR 51%–75%: HR 2.57, *p* < 0.0001
Mavaddat et al. [25], 2014	RCT	MMSE	81.5	238	54.6	VKAs, Antiplatelets	Warfarin was associated with cognitive improvement at 33 months compared to aspirin.	HR (95% CI): 1.48 (0.56, 3.91)
van Deelen et al. [34], 2012	Retrospective Cohort	MMSE	79.1	152	55.9	VKAs	MMSE score <23 linked to insufficient INR.	OR (95% CI): 2.57 (1.02, 6.48)
							Consistently no discernible difference between groups of patients with INR <2.0.	OR (95% CI): 1.03 (0.39, 2.67)
Flaker et al. [29], 2010	Prospective Cohort	MMSE	71	2510	65.5	VKAs	Baseline MMSE significantly affects INR control. Below-median TTR was linked to a modest cognitive decline.	1-point decline in the MMSE (30 to 25) = 1-point reduction in TTR.
Barber et al. [31], 2004	Prospective Cohort	TICSm, IQCODE	72	218	44.5	VKAs	Negative association with warfarin vs. non-warfarin use.	OR (95% CI): 0.52 (0.26, 1.07).

ADAS = Alzheimer’s Disease Assessment Scale; DAD = Disability Assessment for Dementia; ICD = International Classification of Diseases; SPMSQ = Short Portable Mental Status Questionnaire; TICSm = The Modified Telephone Interview for Cognitive Status; IQCODE = Informant Questionnaire on Cognitive Decline In The Elderly; OR = odds ratio; HR = hazard ratio; CI = confidence interval, NA: Not applicable.

## References

[1] Dietzel J., Haeusler K.G., Endres M. (2018). Does atrial fibrillation cause cognitive decline and dementia?. EP Eur..

[2] Santos C.Y., Snyder P.J., Wu W.C., Zhang M., Echeverria A., Alber J. (2017). Pathophysiologic relationship between Alzheimer’s disease, cerebrovascular disease, and cardiovascular risk: A review and synthesis. Alzheimers Dement..

[3] Diener H.C., Hart R.G., Koudstaal P.J., Lane D.A., Lip G.Y.H. (2019). Atrial Fibrillation and Cognitive Function: JACC Review Topic of the Week. J. Am. Coll. Cardiol..

[4] Ding M., Qiu C. (2018). Atrial Fibrillation, Cognitive Decline, and Dementia: An Epidemiologic Review. Curr. Epidemiol. Rep..

[5] Blum S., Conen D. (2023). Mechanisms and Clinical Manifestations of Cognitive Decline in Atrial Fibrillation Patients: Potential Implications for Preventing Dementia. Can. J. Cardiol..

[6] Chinta V., Askandar S., Nanda A., Sharma A., Abader P., Kabra R., Khouzam R.N. (2019). Atrial Fibrillation and Deterioration in Cognitive Function. Curr. Probl. Cardiol..

[7] Kalantarian S., Ruskin J.N. (2016). Atrial Fibrillation and Cognitive Decline: Phenomenon or Epiphenomenon?. Cardiol. Clin..

[8] Prins N.D., Van Dijk E.J., den Heijer T., Vermeer S.E., Koudstaal P.J., Oudkerk M., Hofman A., Breteler M.M. (2004). Cerebral white matter lesions and the risk of dementia. Arch. Neurol..

[9] Anstey K.J., von Sanden C., Salim A., O’Kearney R. (2007). Smoking as a risk factor for dementia and cognitive decline: A meta-analysis of prospective studies. Am. J. Epidemiol..

[10] Biessels G.J., Staekenborg S., Brunner E., Brayne C., Scheltens P. (2006). Risk of dementia in diabetes mellitus: A systematic review. Lancet Neurol..

[11] Wendell C.R., Waldstein S.R., Ferrucci L., O’Brien R.J., Strait J.B., Zonderman A.B. (2012). Carotid atherosclerosis and prospective risk of dementia. Stroke.

[12] Soo Y., Zietz A., Yiu B., Mok V.C.T., Polymeris A.A., Seiffge D., Ambler G., Wilson D., Leung T.W.H., Tsang S.F. (2023). Impact of Cerebral Microbleeds in Stroke Patients with Atrial Fibrillation. Ann. Neurol..

[13] Page M.J., McKenzie J.E., Bossuyt P.M., Boutron I., Hoffmann T.C., Mulrow C.D., Shamseer L., Tetzlaff J.M., Akl E.A., Brennan S.E. (2021). The PRISMA 2020 statement: An updated guideline for reporting systematic reviews. Int. J. Surg..

[14] Seong H.J., Lee K., Kim B.H., Son Y.J. (2019). Cognitive impairment is independently associated with non-adherence to antithrombotic therapy in older patients with atrial fibrillation. Int. J. Environ. Res. Public. Health.

[15] Mongkhon P., Fanning L., Lau W.C., Tse G., Lau K.K., Wei L., Kongkaew C., Wong I.C. (2020). Oral anticoagulant and reduced risk of dementia in patients with atrial fibrillation: A population-based cohort study. Heart Rhythm..

[16] Jankowska-Polańska B., Katarzyna L., Lidia A., Joanna J., Dudek K., Izabella U. (2016). Cognitive function and adherence to anticoagulation treatment in patients with atrial fibrillation. J. Geriatr. Cardiol..

[17] Horstmann S., Rizos T., Saribas M., Efthymiou E., Rauch G., Veltkamp R. (2015). Cognitive Impairment is Not a Predictor of Failure to Adhere to Anticoagulation of Stroke Patients with Atrial Fibrillation. Cerebrovasc. Dis..

[18] Caramelli B., Yu P.C., Cardozo F.A.M., Magalhães I.R., Spera R.R., Amado D.K., Escalante-Rojas M.C., Gualandro D.M., Calderaro D., Tavares C.A.M. (2022). Effects of dabigatran versus warfarin on 2-year cognitive outcomes in old patients with atrial fibrillation: Results from the GIRAF randomized clinical trial. BMC Med..

[19] Bunch T.J., May H., Cutler M., Woller S.C., Jacobs V., Stevens S.M., Carlquist J., Knowlton K.U., Muhlestein J.B., Steinberg B.A. (2022). Impact of anticoagulation therapy on the cognitive decline and dementia in patients with non-valvular atrial fibrillation (cognitive decline and dementia in patients with non-valvular atrial fibrillation [CAF] trial). J. Arrhythmia.

[20] Friberg L., Rosenqvist M. (2018). Less dementia with oral anticoagulation in atrial fibrillation. Eur. Heart J..

[21] Jacobs V., May H.T., Bair T.L., Crandall B.G., Cutler M.J., Day J.D., Mallender C., Osborn J.S., Stevens S.M., Weiss J.P. (2016). Long-Term Population-Based Cerebral Ischemic Event and Cognitive Outcomes of Direct Oral Anticoagulants Compared With Warfarin Among Long-term Anticoagulated Patients for Atrial Fibrillation. Am. J. Cardiol..

[22] Cadogan S.L., Powell E., Wing K., Wong A.Y., Smeeth L., Warren-Gash C. (2021). Anticoagulant prescribing for atrial fibrillation and risk of incident dementia. Heart.

[23] Field T.S., Weijs B., Curcio A., Giustozzi M., Sudikas S., Katholing A., Wallenhorst C., Weitz J.I., Cohen A.T., Martinez C. (2019). Incident Atrial Fibrillation, Dementia and the Role of Anticoagulation: A Population-Based Cohort Study. Thromb. Haemost..

[24] Petroni R., Magnano R., Pezzi L., Petroni A., Di Mauro M., Mattei A., Fiasca F., Angelone A.M., Gallina S., Penco M. (2020). Analysis of Risk Factors Independently Associated with Cognitive Impairment in Patients with Permanent Atrial Fibrillation: A Cross-sectional Observational Study. J. Stroke Cerebrovasc. Dis..

[25] Mavaddat N., Roalfe A., Fletcher K., Lip G.Y., Hobbs F.R., Fitzmaurice D., Mant J. (2014). Warfarin versus aspirin for prevention of cognitive decline in atrial fibrillation: Randomized controlled trial (birmingham atrial fibrillation treatment of the aged study). Stroke.

[26] Wong C.K., Huang D., Zhou M., Hai J., Yue W.S., Li W.-H., Yin L.-X., Zuo M.-L., Feng Y.Q., Tan N. (2022). Antithrombotic therapy and the risk of new-onset dementia in elderly patients with atrial fibrillation. Postgrad. Med. J..

[27] Annweiler C., Ferland G., Barberger-Gateau P., Brangier A., Rolland Y., Beauchet O. (2015). Vitamin K antagonists and cognitive impairment: Results from a cross-sectional pilot study among geriatric patients. J. Gerontol. A Biol. Sci. Med. Sci..

[28] Madhavan M., Hu T.Y., Gersh B.J., Roger V.L., Killian J., Weston S.A., Graff-Radford J., Asirvatham S.J., Chamberlain A.M. (2018). Efficacy of Warfarin Anticoagulation and Incident Dementia in a Community-Based Cohort of Atrial Fibrillation. Mayo Clin. Proc..

[29] Flaker G.C., Pogue J., Yusuf S., Pfeffer M.A., Goldhaber S.Z., Granger C.B., Anand I.S., Hart R., Connolly S.J. (2010). Cognitive function and anticoagulation control in patients with atrial fibrillation. Circ. Cardiovasc. Qual. Outcomes.

[30] Gorzelak-Pabiś P., Zyzak S., Krewko Ł., Broncel M. (2016). Assessment of the mean time in the therapeutic INR range and the SAME-TT2R2 score in patients with atrial fibrillation and cognitive impairment. Pol. Arch. Med..

[31] Barber M., Tait R.C., Scott J., Rumley A., Lowe G.D.O., Stott D.J. (2004). Dementia in subjects with atrial fibrillation: Hemostatic function and the role of anticoagulation. J. Thromb. Haemost..

[32] Jacobs V., Woller S.C., Stevens S., May H.T., Bair T.L., Anderson J.L., Crandall B.G., Day J.D., Johanning K., Long Y. (2014). Time outside of therapeutic range in atrial fibrillation patients is associated with long-term risk of dementia. Heart Rhythm..

[33] Bunch T.J., May H.T., Bair T.L., Crandall B.G., Cutler M.J., Day J.D., Jacobs V., Mallender C., Osborn J.S., Stevens S.M. (2016). Atrial Fibrillation Patients Treated with Long-Term Warfarin Anticoagulation Have Higher Rates of All Dementia Types Compared with Patients Receiving Long-Term Warfarin for Other Indications. J. Am. Heart Assoc.

[34] Van Deelen B.A.J., Van Den Bemt P.M.L.A., Egberts T.C.G., Van’t Hoff A., Maas H.A.A.M. (2005). Cognitive impairment as determinant for sub-optimal control of oral anticoagulation treatment in elderly patients with atrial fibrillation. Drugs Aging.

[35] Malavasi V.L., Zoccali C., Brandi M.C., Micali G., Vitolo M., Imberti J.F., Mussi C., Schnabel R.B., Freedman B., Boriani G. (2021). Cognitive impairment in patients with atrial fibrillation: Implications for outcome in a cohort study. Int. J. Cardiol..

[36] Mongkhon P., Naser A.Y., Fanning L., Tse G., Lau W.C., Wong I.C., Kongkaew C. (2019). Oral anticoagulants and risk of dementia: A systematic review and meta-analysis of observational studies and randomized controlled trials. Neurosci. Biobehav. Rev..

[37] Jacobs L.G., Billett H.H., Freeman K., Dinglas C., Jumaquio L. (2009). Anticoagulation for stroke prevention in elderly patients with atrial fibrillation, including those with falls and/or early-stage dementia: A single-center, retrospective, observational study. Am. J. Geriatr. Pharmacother..

[38] Ott A., Breteler M.M., de Bruyne M.C., van Harskamp F., Grobbee D.E., Hofman A. (1997). Atrial fibrillation and dementia in a population-based study. The Rotterdam Study. Stroke.

[39] Thacker E.L., McKnight B., Psaty B.M., Longstreth W., Sitlani C.M., Dublin S., Arnold A.M., Fitzpatrick A.L., Gottesman R.F., Heckbert S.R. (2013). Atrial fibrillation and cognitive decline: A longitudinal cohort study. Neurology.

[40] Konno S., Meyer J.S., Terayama Y., Margishvili G.M., Mortel K.F. (1997). Classification, diagnosis and treatment of vascular dementia. Drugs Aging.

[41] Bellmann B., Fiebach J., Guttmann S., Lin T., Haeusler K., Bathe-Peters R., Koehler L., Steffens D., Kasner M., Tscholl V. (2017). Incidence of MRI-detected brain lesions and neurocognitive function after electrical cardioversion in anticoagulated patients with persistent atrial fibrillation. Int. J. Cardiol..

[42] Knecht S., Oelschläger C., Duning T., Lohmann H., Albers J., Stehling C., Heindel W., Breithardt G., Berger K., Ringelstein E.B. (2008). Atrial fibrillation in stroke-free patients is associated with memory impairment and hippocampal atrophy. Eur. Heart J..

[43] Chen L.Y., Lopez F.L., Gottesman R.F., Huxley R.R., Agarwal S.K., Loehr L., Mosley T., Alonso A. (2014). Atrial fibrillation and cognitive decline-the role of subclinical cerebral infarcts: The atherosclerosis risk in communities study. Stroke.

[44] Zeng D., Jiang C., Su C., Tan Y., Wu J. (2019). Anticoagulation in atrial fibrillation and cognitive decline: A systematic review and meta-analysis. Medicine.

[45] de Bruijn R.F.A.G., Heeringa J., Wolters F.J., Franco O.H., Stricker B.H.C., Hofman A., Koudstaal P.J., Ikram M.A. (2015). Association between atrial fibrillation and dementia in the general population. JAMA Neurol..

[46] Kamel H., Okin P.M., Elkind M.S.V., Iadecola C. (2016). Atrial Fibrillation and Mechanisms of Stroke: Time for a New Model. Stroke.

[47] Ezekowitz M.D., James K.E., Nazarian S.M., Davenport J., Broderick J.P., Gupta S.R., Thadani V., Meyer M.L., Bridgers S.L. (1995). Silent cerebral infarction in patients with nonrheumatic atrial fibrillation. The Veterans Affairs Stroke Prevention in Nonrheumatic Atrial Fibrillation Investigators. Circulation.

[48] Feinberg W.M., Seeger J.F., Carmody R.F., Anderson D.C., Hart R.G., Pearce L.A. (1990). Epidemiologic features of asymptomatic cerebral infarction in patients with nonvalvular atrial fibrillation. Arch. Intern. Med..

[49] Gaita F., Corsinovi L., Anselmino M., Raimondo C., Pianelli M., Toso E., Bergamasco L., Boffano C., Valentini M.C., Cesarani F. (2013). Prevalence of silent cerebral ischemia in paroxysmal and persistent atrial fibrillation and correlation with cognitive function. J. Am. Coll. Cardiol..

[50] Bretzman J.P., Tseng A.S., Graff-Radford J., Lee H.-C., Asirvatham S.J., Mielke M.M., Knopman D.S., Petersen R.C., Jack C.R., Vemuri P. (2022). Silent cerebral infarcts in patients with atrial fibrillation: Clinical implications of an imaging-adjusted CHA2DS2-VASc score. Cardiol. J..

[51] Saito T., Kawamura Y., Tanabe Y., Asanome A., Takahashi K., Sawada J., Katayama T., Sato N., Aizawa H., Hasebe N. (2014). Cerebral microbleeds and asymptomatic cerebral infarctions in patients with atrial fibrillation. J. Stroke Cerebrovasc. Dis. Off. J. Natl. Stroke Assoc..

[52] Corica B., Romiti G.F., Raparelli V., Cangemi R., Basili S., Proietti M. (2022). Epidemiology of cerebral microbleeds and risk of adverse outcomes in atrial fibrillation: A systematic review and meta-analysis. Europace.

[53] Kalantarian S., Stern T.A., Mansour M., Ruskin J.N. (2013). Cognitive impairment associated with atrial fibrillation: A meta-analysis. Ann. Intern. Med..

[54] Graff-Radford J., Madhavan M., Vemuri P., Rabinstein A.A., Cha R.H., Mielke M.M., Kantarci K., Lowe V., Senjem M.L., Gunter J.L. (2016). Atrial fibrillation, cognitive impairment, and neuroimaging. Alzheimers Dement. J. Alzheimers Assoc..

[55] Cheng W., Liu W., Li B., Li D. (2018). Relationship of Anticoagulant Therapy With Cognitive Impairment Among Patients With Atrial Fibrillation: A Meta-Analysis and Systematic Review. J. Cardiovasc. Pharmacol..

[56] Wang Y., Kong M.C., Lee L.H., Ng H.J., Ko Y. (2014). Knowledge, satisfaction, and concerns regarding warfarin therapy and their association with warfarin adherence and anticoagulation control. Thromb. Res..

[57] Moffitt P., Lane D.A., Park H., O’Connell J., Quinn T.J. (2016). Thromboprophylaxis in atrial fibrillation and association with cognitive decline: Systematic review. Age Ageing.

[58] Orlandi M., Dover D.C., Sandhu R.K., Hawkins N.M., Kaul P., McAlister F.A. (2022). The Introduction of Direct Oral Anticoagulants Has Not Resolved Treatment Gaps for Frail Patients With Nonvalvular Atrial Fibrillation. Can. J. Cardiol..

[59] Gerstenecker A., Norling A.M., Jacob A., Lazar R.M. (2023). Silent Brain Infarction, Delirium, and Cognition in Three Invasive Cardiovascular Procedures: A Systematic Review. Neuropsychol. Rev..

[60] Herm J., Schurig J., Martinek M.R., Höltgen R., Schirdewan A., Kirchhof P., Wieczorek M., Pürerfellner H., Heuschmann P.U., Fiebach J.B. (2019). MRI-detected brain lesions in AF patients without further stroke risk factors undergoing ablation—A retrospective analysis of prospective studies. BMC Cardiovasc. Disord..

